# Effectiveness and safety of mepolizumab in combination with corticosteroids in patients with eosinophilic granulomatosis with polyangiitis

**DOI:** 10.1186/s13075-021-02462-6

**Published:** 2021-03-16

**Authors:** Masanobu Ueno, Ippei Miyagawa, Kazuhisa Nakano, Shigeru Iwata, Kentaro Hanami, Shunsuke Fukuyo, Satoshi Kubo, Yusuke Miyazaki, Akio Kawabe, Hiroko Yoshinari, Shingo Nakayamada, Yoshiya Tanaka

**Affiliations:** The First Department of Internal Medicine, School of Medicine, University of Occupational and Environmental Health, Japan, 1-1 Iseigaoka, Kitakyushu, 807-8555 Japan

**Keywords:** Eosinophilic granulomatosis with polyangiitis, Corticosteroid, Mepolizumab, Treatment

## Abstract

**Background:**

Mepolizumab (MPZ), an anti-interleukin-5 antibody, is effective for the treatment of eosinophilic granulomatosis with polyangiitis (EGPA). However, its effectiveness has not been adequately evaluated in real-world clinical practice. In this study, we assessed the effectiveness and safety of MPZ (300 mg) for relapsing/refractory EGPA resistant to corticosteroids (CS) for 1 year in real-world settings.

**Methods:**

We administered MPZ (300 mg) to 16 patients with relapsing/refractory EGPA resistant to CS (Post-MPZ). We also retrospectively collected data from the same patients for the 12 months before the administration of MPZ (Pre-MPZ). The primary endpoint was the 12-month remission rate after MPZ administration and the secondary endpoints were the Birmingham vasculitis activity score (BVAS), vasculitis damage index (VDI), eosinophil counts, changes in concomitant CS doses/concomitant immunosuppressant use, MPZ retention rate, and incidence of adverse events. The clinical course was compared between Pre-MPZ and Post-MPZ.

**Results:**

The 12-month remission rate after the initiation of MPZ was 75%. No change was observed in BVAS, eosinophil count, or concomitant CS dose over time in the Pre-MPZ group, whereas all these parameters were significantly decreased over time in the Post-MPZ group. The number of patients using concomitant immunosuppressant also decreased over time in the Post-MPZ group. VDI did not increase in either group. The MPZ retention rate was 100% and only three patients (18.8%) had infections. Changes in BVAS, eosinophil count, and cumulative concomitant CS dose were significantly lower in the Post-MPZ group than in the Pre-MPZ group. There was no significant difference in the changes in VDI between the groups.

**Conclusion:**

This study demonstrated that MPZ is effective and safe for EGPA. Furthermore, MPZ decreases disease activity, increases remission rate, and has a CS-sparing effect.

## Key messages

MPZ is safe for the treatment of EGPA in real-world clinical practice.

Comparing to Pre-MPZ, MPZ possesses the high remission rate and CS sparing effect.

## Background

Eosinophilic granulomatosis with polyangiitis (EGPA) is a disease that is preceded by asthma or allergic rhinitis. EGPA causes various symptoms owing to vasculitis, including fever and purpura, and increases peripheral eosinophil counts [1, 2]. Corticosteroids (CS) are used in remission induction therapy and maintenance therapy for EGPA. Patients with severe vasculitis symptoms and those who respond poorly to CS are treated with cyclophosphamide (CY), azathioprine (AZ), methotrexate (MTX), cyclosporine (CsA)/tacrolimus (TAC), or intravenous immunoglobulin (IVIG). However, EGPA often relapses during CS dose reduction; hence, CS dose reduction is often challenging [3–5].

In recent years, mepolizumab (MPZ), an anti-interleukin-5 (IL-5) monoclonal antibody, has been reported to extend the remission period of EGPA and reduce the CS dose required [6]. Although MPZ was listed in the National Health Insurance drug price list for the treatment of EGPA in Japan in 2018, its effectiveness and safety have not been adequately evaluated in real-world clinical practice. Although the dose of MPZ administered in previous studies was 100 mg/month, which is the same dose used for the treatment of bronchial asthma, an MPZ dose of 300 mg is used in some countries, including Japan. Thus, we investigated the effectiveness and safety of MPZ at a dose of 300 mg/month in a real-world setting.

## Methods

### Patients

In this study, MPZ (300 mg) was administered to 16 patients with relapsing or refractory EGPA who were receiving the standard of care, mainly CS [7, 8]. In Japan, the use of MPZ is allowed when the effect of CS therapy is insufficient. All patients used in this study met this criterion. All patients were diagnosed with EGPA according to the diagnostic criteria for EGPA, as proposed by the Japanese Ministry of Health, Labour and Welfare, and met the classification criteria of the American College of Rheumatology [9] (Table 1, Supplementary Table 1). Patients in remission and those with relapsing or refractory EGPA were defined as follows according to the criteria of the Mepolizumab Treatment in Relapsing or Refractory EGPA trial [6]: patients with remission had a Birmingham vasculitis activity score (BVAS) of 0 and were treated with oral CS at a dose of ≤ 4 mg/day; patients with relapsing EGPA had an increased oral CS dose, started concomitant immunosuppressive therapy, had an increased concomitant immunosuppressant dose, had an increased BVAS [10], or had a history of hospitalization; and patients with refractory EGPA experienced no relapse and achieved no remission within the last 1 year.

**Table 1:** Baseline characteristic of 16 patients with eosinophilic granulomatosis with polyangiitis

	Pre-MPZ (***n*** = 16)	Post–MPZ (***n*** = 16)	***P*** value^*^
Clinical manifestations at diagnosis, *n* (%)	Asthma 16 (100), general 10 (62.5), cutaneous 8 (50.0), ENT 5 (31.3), chest 8 (50.0), cardiomyopathy 3 (18.8), abdominal 1 (6.3), neuropathy 8 (50.0), ANCA positive status 5 (31.3), biopsy findings 12 (75)		
Male/female/age at MPZ introduction	7/9/61.5 [53.3–70.5]		
Disease duration (months) at MPZ introduction	54 [22–144]		
Treatment history, *n* (%)	CS pulse 2 (12.5), high-dose CS 14 (87.5), low-dose CS 2 (12.5), IVCY 9 (56.3), IVIG 6 (37.5), RTX 1 (6.3), MTX 6 (37.5), AZ 12 (75.0), TAC 1 (6.3)		
Relapsing/refractory/remission, *n* (%)	4 (25.0)/11 (68.7)/1 (6.3)	10 (62.5)/6 (37.5)/0 (0)	ND
Concomitant CS dose (PSL mg/day)	8.0 [5.0–11.5]	6.5 [2.6–10.0]	0.2012
Concomitant CS < 4 mg/day (PSL), *n* (%)	3 (18.8)	5 (31.3)	0.1573
Concomitant immunosuppressant, *n* (%)	AZ 6 (37.5), MTX 5 (31.3), TAC 1 (6.3)	AZ 6 (37.5), MTX 4 (25.0), TAC 1 (6.3)	
Without immunosuppressant, *n* (%)	6 (37.5)	7 (43.8)	0.3173
BVAS	0 [0–2.0]	1.0 [0–3.8]	0.1084
BVAS > 0, *n* (%)	4 (25.0)	8 (50.0)	0.3173
BVAS items	Asthma 2 (12.5), sinonasal 2 (12.5), chest 1 (6.3)	Asthma 6 (37.5), general 1 (6.3), cutaneous 2 (12.5), sinonasal 2 (12.5), chest 3 (18.8),	
VDI	3.5 [3.0–4.8]	4.0 [3.0–5.8]	0.5577
VDI items	Chronic bronchial asthma 16 (100), chronic respiratory failure 1 (6.3), abnormal respiratory function 7 (43.8), old myocardial infarction 2 (12.5), cardiomyopathy 2 (12.5), low vision 1 (6.3), chronic sinusitis 6 (37.5), deafness 3 (18.8), peripheral neuropathy 8 (50.0), diabetes 4 (25), hypertension 4 (25), osteoporosis 5 (31.3), other 3 (18.8)	Chronic bronchial asthma 16 (100), chronic respiratory failure 1 (6.3), abnormal respiratory function 8 (50.0), old myocardial infarction 2 (12.5), cardiomyopathy 2 (12.5), low vision 1 (6.3), chronic sinusitis 7 (43.8), deafness 3 (18.8), peripheral neuropathy 8 (50.0), diabetes 4 (25), hypertension 4 (25), osteoporosis 5 (31.3), other 5 (31.3)	
ANCA-positive status, *n* (%)	0 (0)	1 (6.3)	ND
Absolute eosinophil count (/μL)	178.6 [48.7–370.2]	183 [60.0–2479]	0.1591
CRP (mg/dL)	0.06 [0.03–0.09]	0.09 [0.05–0.24]	0.0593

*CS* corticosteroid (prednisolone or equivalent), *IVCY* cyclophosphamide pulse therapy i.v., *RTX* rituximab, *MTX* methotrexate, *AZ* azathioprine, *TAC* tacrolimus, *BVAS* Birmingham Vasculitis Activity Score, *VDI* vasculitis damage index, *ND* not detected by McNemar test. Data are shown by median [quartile] or *n* (%). *P* values were determined by McNemar test or Wilcoxon signed-rank test. *P < 0.05: Pre-MPZ (*n* = 16) vs. Post-MPZ (*n* = 16)

The patients were followed up for 12 months after the introduction of MPZ at our hospital and affiliated institutions, during the period between the domestic introduction of MPZ in May 2018 until August 2020 (Post-MPZ). Additionally, we retrospectively collected data from the same patients for the 12 months before the initiation of MPZ therapy (Pre-MPZ). All patients received maintenance therapy according to the standard of care. The standard of care in this study was defined as treatment with CS, intravenous CY, intravenous immunoglobulin, azathioprine, MTX, or CsA/TAC. The Human Ethics Review Committee of our university reviewed and approved this study (No. H27-014). We also complied with the Declaration of Helsinki. All participants provided informed consent prior to inclusion in the study. Details that might disclose the identity of the study subjects were omitted.

### Clinical measurement

This study was a multicenter and ambispective cohort study in which MPZ at a dose of 300 mg/month was administered to 16 patients with relapsing or refractory EGPA to investigate the effectiveness and safety of MPZ over 1 year.

The primary endpoint was the remission rate. The secondary endpoints were the BVAS (overall and for each item), vasculitis damage index (VDI) (overall and for each item) [11, 12], eosinophil counts, daily and cumulative concomitant CS doses, presence or absence of changes/addition of immunosuppressant(s), MPZ retention rate, and incidence of adverse events. Additionally, the reduction in BVAS, change in VDI, reduction in peripheral eosinophil counts, and cumulative concomitant CS doses were compared between the 1-year period before the initiation of MPZ therapy (Pre-MPZ; month − 12 to month 0) and 1-year period after the initiation of MPZ (Post-MPZ; month 0 to month 12). To express the results of the two groups synchronously, the original timeframes Pre-MPZ (− 12 (baseline), − 11, − 9, and − 6 months) are represented as 0 (baseline), 1, 3, and 6 months, respectively.

### Measurement of serum concentration of IL-5

The serum concentration of IL-5 at the baseline (before MPZ initiation) was measured using the enzyme-linked immunosorbent assay (ELISA) (R&D SYSTEMS Human IL-5 Duo Set ELISA, P249454) and compared with that of six age- and sex-matched healthy controls.

### Statistical analysis

Data are expressed as the median (interquartile range). For statistical analysis, data from cases in which MPZ was discontinued or the disease relapsed were complemented using the last observation carried forward method. Differences between groups (Post-MPZ vs. Pre-MPZ) and between data measured at the baseline and each observation point (Post-MPZ: month 0 vs. month 1, 3, 6, 12) were compared using the Wilcoxon signed-rank test or McNemar test. Differences in the serum IL-5 concentration between the patients and healthy controls were compared using the Mann-Whitney *U* test.

The timeframes of both groups were represented synchronously and compared [− 12 months (baseline) in the Pre-MPZ corresponded to 0 months (baseline) in the Post-MPZ group]. All reported *P* values are two-sided. Remission was defined as a BVAS score of 0 and CS less than 4 mg/day. All analyses were conducted using JMP version 14.0.0 (SAS Institute Inc.). The post hoc power of this study for the comparison between month 0 and the other observation points in the Post-MPZ group was 0.37 for BVAS, 0.99 for VDI, 0.36 for absolute eosinophil count, and 0.76 for concomitant CS dose (*α* error, 0.05; 1-β error, post hoc power).

## Results

### Patient background

The characteristics of the patients are shown in Table 1. The characteristics of each patient at the time of EGPA diagnosis are shown in Supplementary Table 1 and those at the time of MPZ therapy initiation are shown in Supplementary Table 2. At the time of MPZ therapy initiation, the median age [interquartile range] of the 16 patients with EGPA was 61.5 [53.3–70.5] years and the disease duration was 54 [22–144] months. Regarding medical history, all patients were treated with CS. The Pre-MPZ group included four patients with relapsing EGPA, 11 with refractory EGPA, and one in remission, whereas the Post-MPZ group included 10 patients with relapsing EGPA and six with refractory EGPA. No statistically significant differences were observed in the concomitant CS dose or the rate of concomitant immunosuppressant use between the groups. There were also no statistically significant differences in BVAS, VDI, positivity rate for anti-neutrophil cytoplasmic antibody, eosinophil counts, or C-reactive protein level between the groups. The IL-5 concentration before MPZ therapy initiation was 1.88 [0.28–8.95] pg/mL, which was significantly higher than that in the six age- and sex-matched healthy controls (0.027 [0.003–0.55], *P* = *0.0063 using Mann-Whitney *U* test; Supplementary Fig. 1).

### Effectiveness of MPZ

**Fig. 1:**
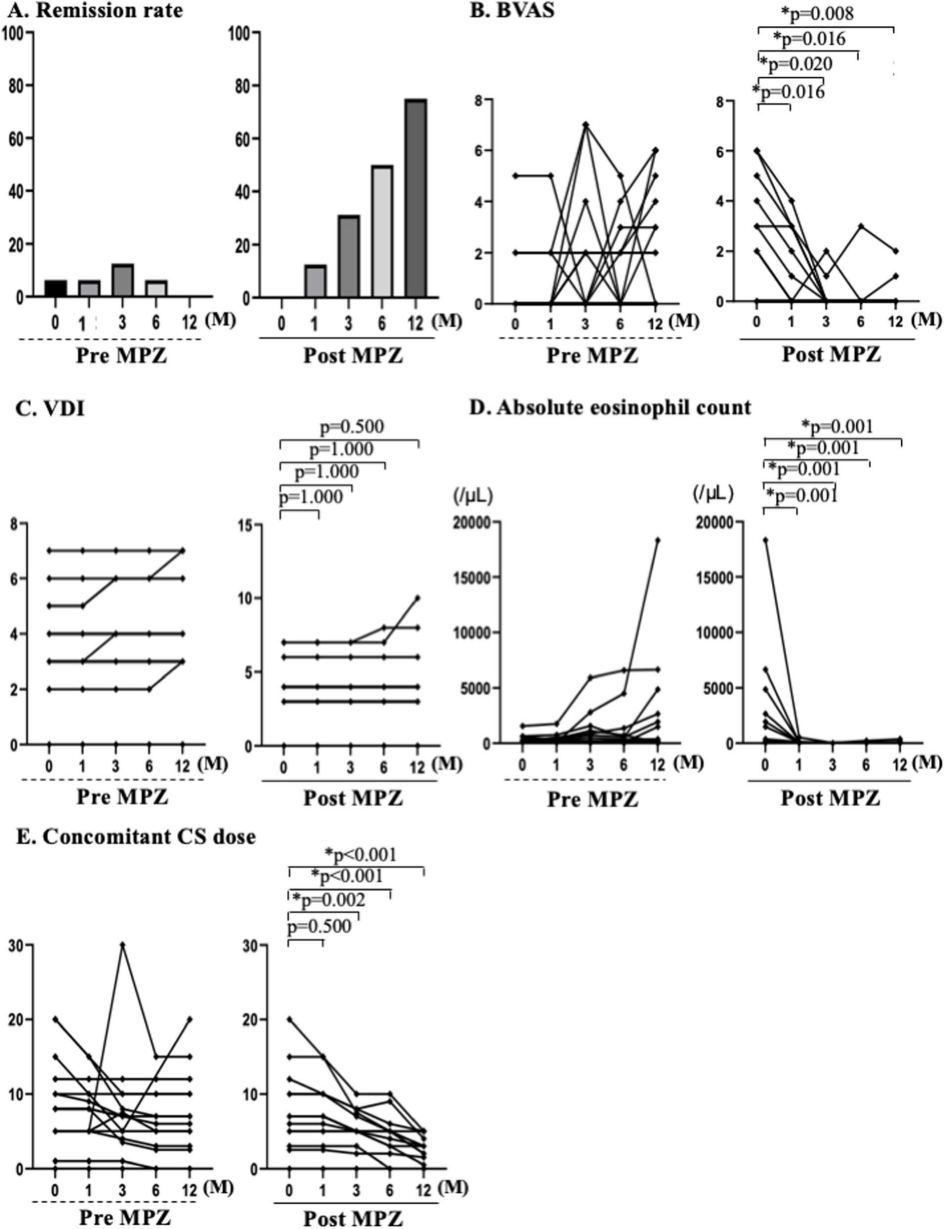
Comparison of effectiveness between the Pre-MPZ and the Post-MPZ. **a** Remission rates. **b** BVAS. **c** VDI. **d** Eosinophil counts. **e** Concomitant CS doses. BVAS, Birmingham vasculitis activity score; CS, corticosteroid; VDI, vasculitis damage index. *P* values were determined by Wilcoxon signed-rank test. *P < 0.05: baseline (month 0) vs. each observation points (month 0,1, 3, 6, and 12)

The remission rates (the primary endpoint) were 6.3% (1/16 patients) at month 1, 12.5% (2/16 patients) at month 3, 6.3% (1/16 patients) at month 6, and 0% at month 12 in the Pre-MPZ group. The corresponding rates in the Post-MPZ group were 12.5% (2/16 patients) at month 1, 31.3% (5/16 patients) at month 3, 50.0% (8/16 patients) at month 6, and 75.0% (12/16 patients) at month 12. In this group, the remission rate increased at month 12 (Fig. 1a).

**Fig. 2:**
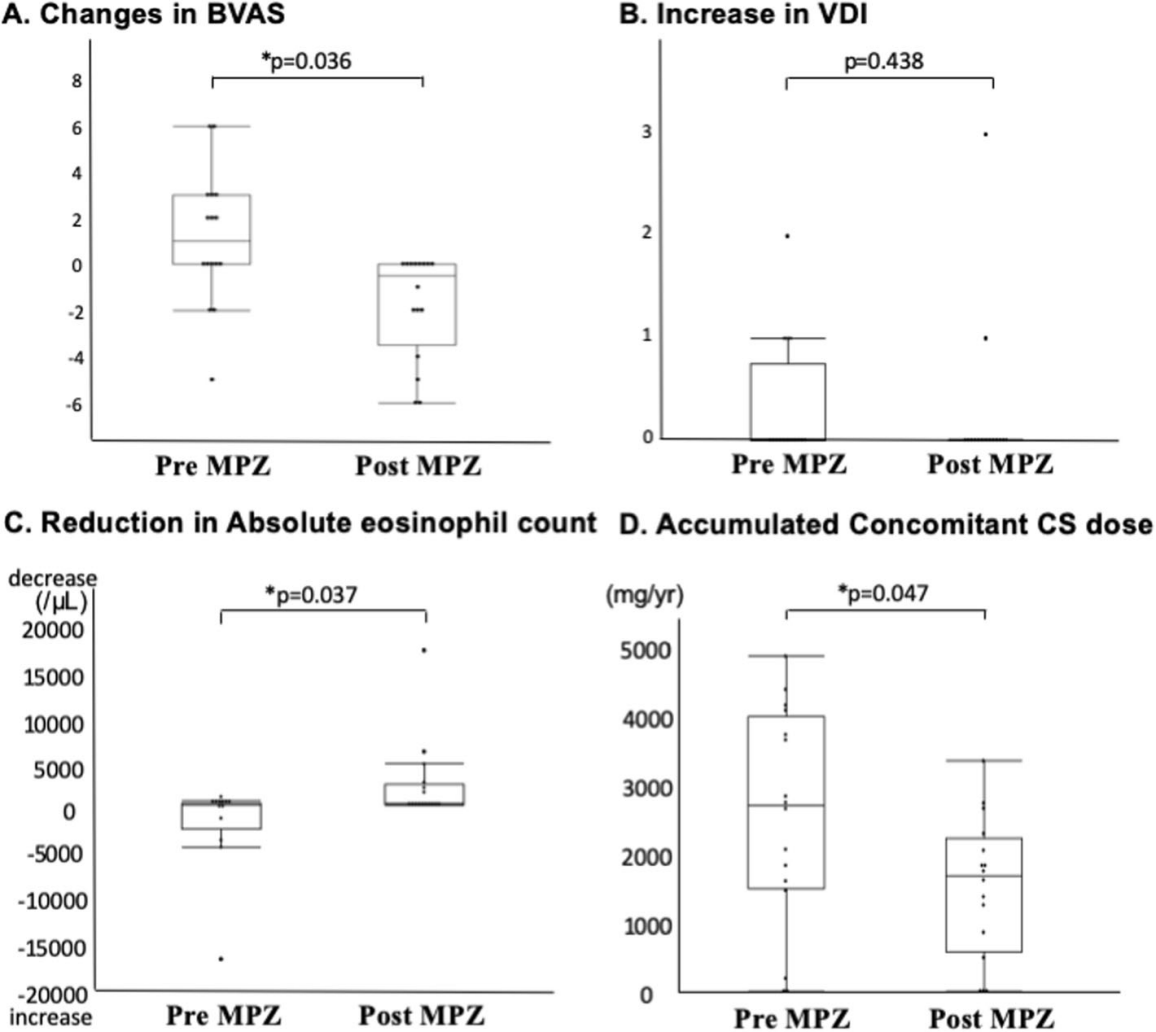
Comparison of changes of each item between the 12-month period in Pre-MPZ and Post-MPZ. **a** Changes in BVAS, **b** increase in VDI, **c** reduction in peripheral eosinophil counts (/μL), and **d** accumulated concomitant CS dose (mg/year). BVAS, Birmingham vasculitis activity score; CS, corticosteroid; VDI, vasculitis damage index. *P* values were determined by Wilcoxon signed-rank test. *P < 0.05: Pre-MPZ vs. Post-MPZ

In the Pre-MPZ group, the BVASs were 0 [0–2.0] at month 1, 0 [0–2.0] at month 3, 0 [0–2.0] at month 6, and 1.0 [0–3.8] at month 12. In the Post-MPZ group, the BVASs were 0 [0–2.8] at month 1, 0 [0–0] at month 3, 0 [0–0] at month 6, and 0 [0–0] at month 12. The BVASs at month 1 and afterward significantly decreased from the BVAS at month 0 (Fig. 1b). The decrease in BVAS during the 1-year period in the Post-MPZ group was 0.5 [0–3.5], which was significantly higher than that in the Pre-MPZ group (− 1.0 [− 3.0–0]; Fig. 2a).

Based on the changes in BVASs for each item, respiratory symptoms were exacerbated in the Pre-MPZ group but improved immediately after the initiation of MPZ therapy. The number of patients with symptoms decreased from 11/16 to 2/16 after 1 year of treatment. Ear, nose, and throat symptoms also improved, as the number of patients with these symptoms decreased from 6/16 to 3/16 patients, 1 year after the initiation of MPZ therapy. In contrast, neuropathy did not improve in either the Post-MPZ or Pre-MPZ groups. In the Post-MPZ group, no organ dysfunction was exacerbated at month 12 (Table 2).

**Table 2:** Changes in organ damage before and after the introduction of MPZ

	Pre-MPZ				Post-MPZ				
	-12 M	-11 M	-9 M	-6 M	0 M	1 M	3 M	6 M	12 M
General symptoms	0	0	1 (6.3%)	1 (6.3%)	1 (6.3%)	1 (6.3%)	0	1 (6.3%)	0
Cutaneous manifestations	1 (6.3%)	1 (6.3%)	1 (6.3%)	1 (6.3%)	2 (12.5%)	2 (12.5%)	0	1 (6.3%)	0
ENT manifestations	6 (37.5%)	6 (37.5%)	6 (37.5%)	6 (37.5%)	6 (37.5%)	6 (37.5%)	6 (37.5%)	4 (25.0%)	3 (18.8%)
Chest manifestations	6 (37.5%)	6 (37.5%)	8 (50.0%)	8 (50.0%)	11 (68.8%)	5 (31.3%)	2 (12.5%)	2 (12.5%)	2 (12.5%)
Nervous system manifestations	7 (45.8%)	7 (45.8%)	7 (45.8%)	8 (50.0%)	8 (50.0%)	8 (50.0%)	7 (43.8%)	7 (43.8%)	7 (43.8%)

*ENT* ear, nose, throat

In the Pre-MPZ group, the VDI scores were 3.5 [3.0–4.8] at month 1, 4.0 [3.0–5.5] at month 3, 4.0 [3.0–5.5] at month 6, and 4.0 [3.0–5.5] at month 12. In the Post-MPZ group, the VDI scores were 4.0 [3.0–5.5] at month 1, 4.0 [3.0–5.5] at month 3, 4.0 [3.0–5.5] at month 6, and 4.0 [3.0–5.5] at month 12, with no significant changes (Fig. 1c). The increase in the VDI score during the 1-year period was 0 [0–0.8] in the Pre-MPZ and 0 [0–0] in the Post-MPZ groups, with no significant difference between the groups (Fig. 2b).

In the Pre-MPZ group, the eosinophil counts were 280.4 [63.2–426.8]/μL at month 1, 217.8 [93.3–1354.2]/μL at month 3, and 293.9 [39.1–880.7]/μL at month 6. In the Post-MPZ group, the eosinophil counts were 54.8 [10.6–99.8]/μL at month 1, 25.2 [12.8–53.9]/μL at month 3, 29.4 [9.63–42.5]/μL at month 6, and 28.8 [20.5–68.0] at month 12, with a significant reduction from month 1 onwards (Fig. 1d). The reduction in the eosinophil counts during the 1-year period in the Post-MPZ group was 146.2 [9.88–2449.9], which was significantly higher than that in the Pre-MPZ group (− 8.8 [− 2927.4–175.4]; Fig. 2c).

The changes in the concomitant CS dose in each patient are shown in Table 3. In the Pre-MPZ group, the concomitant CS doses were 8.0 [5.0–10.0] mg/day at month 1, 7.0 [3.5–10.0] mg/day at month 3, 6.5 [2.6–10.0] mg/day at month 6, and 6.0 [2.6–10.0] mg/day at month 12. In the Post-MPZ group, the concomitant CS doses were 6.5 [2.6–10.0] mg/day at month 1, 5.0 [2.3–7.4] mg/day at month 3, 4.5 [0.5–5.0] mg/day at month 6, and 2.5 [0.1–3.8] mg/day at month 12, with a significant reduction from month 3 onwards (Fig. 1e). The concomitant CS dose was significantly lower in the Post-MPZ group (1655 [570.0–2190.0] mg/year) than in the Pre-MPZ group (2665 [1473.8–3993.8] mg/year; Fig. 2d). The changes in the use of immunosuppressants are shown in Table 4. The number of patients using concomitant immunosuppressant(s) reduced from 10 to nine patients at 1 year in the Pre-MPZ group and from nine to five patients in the Post-MPZ group.

**Table 3:** Changes in the concomitant corticosteroids use before and after the introduction of MPZ (PSL mg/day)

Case No.	Pre-MPZ				Post-MPZ				
	− 12 months	− 11 months	− 9 months	− 6 months	0 months	1 months	3 months	6 months	12 months
1	5 mg	5 mg	30 mg	15 mg	15 mg	15 mg	10 mg	10 mg	5 mg
2	5 mg	5 mg	7.5 mg	5 mg	5 mg	5 mg	5 mg	5 mg	5 mg
3	8 mg	8 mg	3.5 mg	2.5 mg	2.5 mg	2.5 mg	2 mg	2 mg	1.5 mg
4	12 mg	12 mg	12 mg	12 mg	12 mg	10 mg	8 mg	9 mg	4 mg
5	8 mg	8 mg	7 mg	7 mg	7 mg	7 mg	5 mg	5 mg	3 mg
6	1 mg	1 mg	1 mg	0 mg	0 mg	0 mg	0 mg	0 mg	0 mg
7	0 mg	0 mg	0 mg	0 mg	0 mg	0 mg	0 mg	0 mg	0 mg
8	10 mg	9 mg	7 mg	6 mg	6 mg	6 mg	5 mg	3 mg	0.5 mg
9	5 mg	5 mg	4 mg	3 mg	3 mg	3 mg	3 mg	0 mg	0 mg
10	15 mg	10 mg	5 mg	35 mg	20 mg	15 mg	7.5 mg	5 mg	3 mg
11	8 mg	8 mg	7 mg	7 mg	7 mg	7 mg	5 mg	5 mg	2 mg
12	20 mg	15 mg	10 mg	10 mg	10 mg	10 mg	7 mg	5 mg	2 mg
13	10 mg	10 mg	10 mg	10 mg	10 mg	10 mg	8 mg	6 mg	5 mg
14	5 mg	5 mg	5 mg	5 mg	5 mg	5 mg	5 mg	3 mg	3 mg
15	20 mg	15 mg	8 mg	7 mg	7 mg	7 mg	5 mg	4 mg	3 mg
16	0 mg	0 mg	0 mg	0 mg	0 mg	0 mg	0 mg	0 mg	0 mg

**Table 4:** Changes in the concomitant immunosuppressants use before and after the introduction of MPZ

Case No.	Pre-MPZ				Post-MPZ				
	− 12 months	− 11 months	− 9 months	− 6 months	0 months	1 months	3 months	6 months	12 months
1	MTX 8 mg	MTX 8 mg	None	None	None	None	None	None	None
2	MTX 8 mg+AZ 125 mg	MTX 8 mg+AZ 125 mg	MTX 8 mg+AZ 125 mg	MTX 8 mg+AZ 125 mg	MTX 8 mg+AZ 125 mg	AZ125 mg	AZ125 mg	AZ125 mg	AZ100 mg
3	AZ50 mg	AZ50 mg	AZ 50 mg	AZ 50 mg	AZ 50 mg	AZ 50 mg	AZ 50 mg	AZ 50 mg	AZ 50 mg
4	None	None	AZ 50 mg	None	None	None	None	MTX 8 mg	MTX 8 mg
5	MTX 6 mg+AZ 50 mg	MTX 6 mg+AZ 50 mg	MTX 6 mg+AZ 50 mg	MTX 6 mg+AZ 50 mg	MTX 6 mg+AZ 50 mg	AZ 50 mg	AZ 50 mg	AZ 50 mg	AZ 50 mg
6	MTX 16 mg	MTX 16 mg	MTX 16 mg	MTX 16 mg	MTX 16 mg	MTX 16 mg	MTX 10 mg	MTX 4 mg	None
7	None	None	None	None	None	None	None	None	None
8	MTX 12 mg	MTX 12 mg	MTX 12 mg	MTX 12 mg	MTX 12 mg	None	None	None	None
9	AZ 50 mg	AZ 50 mg	AZ 50 mg	AZ 50 mg	AZ 50 mg	None	None	None	None
10	None	None	IVCY	None	None	None	None	None	None
11	TAC 3 mg	TAC 3 mg	TAC 3 mg	TAC 3 mg	TAC 3 mg	None	None	None	None
12	None	None	None	None	None	None	None	None	None
13	AZ 50 mg	AZ 50 mg	AZ 50 mg	AZ 50 mg	AZ 50 mg	AZ 50 mg	AZ 50 mg	AZ 50 mg	AZ 50 mg
14	None	None	None	None	None	None	None	None	None
15	AZ 100 mg	AZ 100 mg	AZ 100 mg	AZ 100 mg	AZ 100 mg	None	None	None	None
16	None	None	None	None	None	None	None	None	None

*IVCY* cyclophosphamide pulse therapy i.v., *MTX* methotrexate, *AZ* azathioprine, *TAC* tacrolimus

### MPZ retention rate and safety

The 1-year MPZ retention rate was 100%. Although three patients had an infection, all patients continued MPZ. Adverse events before and after the initiation of MPZ therapy are shown in Table 5. After MPZ therapy initiation, cases 1 and 11 developed bacterial pneumonia that required hospitalization. Sputum culture identified *Moraxella catarrhalis* in case 1 and *Pseudomonas aeruginosa* in case 11. In both patients, a drip infusion of antibiotics improved their condition. Case 15 also developed bacterial pneumonia. Because the general and respiratory conditions were favorable, the patient received oral antibiotic therapy at the outpatient clinic and improved. All three patients who had an infection after the initiation of MPZ therapy had the same infection within 1 year before initiation. None of the patients had a new infection after the initiation of therapy.

**Table 5:** Adverse events before and after introduction of MPZ

Case No.	Pre-MPZ	Post-MPZ
1	Bacterial pneumonia (hospitalization)	Bacterial pneumonia (hospitalization) Bacterial species: *Moraxella catarrhalis*
2	None	None
3	None	None
4	Drug-induced liver injury	None
5	Sinusitis surgery, bacterial bronchitis	None
6	Bacterial bronchitis, infectious otitis media	None
7	None	None
8	None	None
9	None	None
10	None	None
11	Bacterial pneumonia (hospitalization)	Bacterial pneumonia (hospitalization) Bacterial species: *Pseudomonas aeruginosa*
12	None	None
13	Bacterial pneumonia (hospitalization)	None
14	Bacterial bronchitis	None
15	Bacterial pneumonia	Bacterial pneumonia
16	None	None

## Discussion

The results of this study demonstrated the effectiveness and safety of MPZ for relapsing or refractory EGPA in a real-world setting by comparing the clinical courses before and after the initiation of MPZ therapy. During the 1-year period before MPZ therapy initiation, BVASs increased as CS doses were tapered, although the effectiveness of immunosuppressants in controlling disease activity was inadequate to allow CS dose reduction. Although many patients used AZ as a concomitant immunosuppressant before MPZ therapy initiation in this study, it has been previously reported that AZ is not useful for maintenance therapy [13]. In this study, BVASs and eosinophil counts significantly decreased 1 month after MPZ therapy initiation. The doses of CS and immunosuppressants were also successfully reduced (Figs. 2a, c, d). MPZ was a useful drug for maintenance therapy that exerted a more consistent effect in controlling disease activity than existing immunosuppressants.

Although the 16 patients included in this study had relatively low eosinophil counts (Table 1), the IL-5 levels before MPZ initiation were significantly higher than those of healthy controls. However, MPZ was effective even in patients whose serum concentration of IL-5 was comparable to that of healthy controls. We believe that these results indicate that MPZ is an effective treatment option in patients with relapsing or refractory EGPA, regardless of IL-5 concentration.

The MPZ retention rate was 100%, and the incidence of infections tended to decrease after MPZ initiation (Table 2). These results confirm the safety of MPZ. In particular, a severe infection that required hospitalization was noted only in two patients with a history of infection, and there was no occurrence of a new serious infection. These results can be considered useful. The reduced incidence of infection might be attributable to the significant reduction in concomitant CS doses and the reduced number of patients using concomitant immunosuppressant after MPZ therapy initiation (Fig. 2d; Tables 3 and 4). The long-term oral administration of CS induces infections and various complications, including osteoporosis, diabetes, hypertension, dyslipidemia, and femoral head necrosis. We did not observe a significant increase in VDI scores nor an increased incidence of complications owing to CS after MPZ therapy initiation. Thus, we demonstrated that MPZ therapy was sufficiently effective in controlling disease activity and prevented adverse events induced by CS and immunosuppressants. In the future, it is important that a long-term investigation is conducted to determine whether long-term MPZ therapy allows the dose reduction or discontinuation of CS without relapse and whether VDI increases.

However, this study has important limitations. For example, this study had limited statistical power owing to the small sample size. Additionally, because the Pre-MPZ group was set as the control group to compare the effects of MPZ therapy with, the control group might be inadequate. Moreover, few countries recommend subcutaneous MPZ injection at a dose of 300 mg for EGPA, such as Japan. A strength of this study is that although there are some case reports of the use of MPZ at a dose of 300 mg [14] and a case series of the use of MPZ at 100 mg for the treatment of comorbid asthma [15], no studies have investigated the safety and effectiveness of MPZ at 300 mg in real-world clinical practice. To the best of our knowledge, this is the first study to demonstrate the safety and effectiveness of MPZ at 300 mg in a real-world setting.

## Supplementary Information

**Additional file 1: Supplementary Table 1.** Clinical manifestations, Japanese Ministry of Health, Labor and Welfare criteria items and classification criteria of the American College of Rheumatology criteria at diagnosis.

**Additional file 2: Supplementary Table 2.** Baseline characteristic of 16 Patients with Eosinophilic Granulomatosis with Polyangiitis.

**Additional file 3: Supplementary Fig. 1.** Serum IL-5 concentration of 16 Patients with EGPA before initiating MPZ and health controls (HC group). *P* values were determined by Mann-Whitney’s U test. p* < 0.01: EGPA group (*n* = 16) vs. HC (*n* = 6).

## Data Availability

Not applicable.

## Abbreviations

MPZ: Mepolizumab
EGPA: Eosinophilic granulomatosis with polyangiitis
CS: Corticosteroids
BVAS: Birmingham vasculitis activity score
VDI: Vasculitis damage index
CY: Cyclophosphamide
IVCY: Intravenous cyclophosphamide
AZ: Azathioprine
MTX: Methotrexate
CsA: Cyclosporine
TAC: Tacrolimus
IVIG: Intravenous immunoglobulin
Pre-MPZ: 1-Year period before initiating MPZ therapy
Post-MPZ: 1-Year period after initiating MPZ
