# Supplementary material for: Effectiveness and safety of mepolizumab in combination with corticosteroids in patients with eosinophilic granulomatosis with polyangiitis
Source: Arthritis Res Ther. 2021 Mar 16;23:86. doi: 10.1186/s13075-021-02462-6 (PMC7962235; doi:10.1186/s13075-021-02462-6)
Supplement: Supplementary file 1 — Additional file 1: Supplementary Table 1. Clinical manifestations, Japanese Ministry of Health, Labor and Welfare criteria items and classification criteria of the American College of Rheumatology criteria at diagnosis. [file 13075_2021_2462_MOESM1_ESM.docx]

|  |  |  | **Clinical manifestation** | | | | | | | | | **Japanese Ministry of Health, Labour and Welfare criteria** | | | | | **American College of Rheumatology criteria** | | | | | |
| --- | --- | --- | --- | --- | --- | --- | --- | --- | --- | --- | --- | --- | --- | --- | --- | --- | --- | --- | --- | --- | --- | --- |
| Case  No. | age | sex | Asthma with eosinophilia | General  symptoms | Nervous  system | Lung | ENT | Heart | Palpable purpura | Abdominal | ANCA  positive status | (A)  Bronchial asthma  or Allergic Rhinitis | (B)  Hyper-  eosinophilia | (C)  Symptoms of  vasculitis | (D) Characteristic  clinical progress | (E)  Biopsy findings | (A)  asthma | (B)  Hyper-  eosinophilia | (C)  Mono-  neuropathy | (D)  Lung infiltration | (E)  Paranasal  abnormalities | (F)  Eosinophilic  infiltration |
| 1 | 51 | M | + | + | + | + | - | + | + | - | - | + | + | + | + | + | + | + | + | + | - | + |
| 2 | 43 | F | + | - | - | - | + | - | - | - | + | + | + | + | + | + | + | + | - | - | + | + |
| 3 | 35 | F | + | + | - | + | - | - | + | - | - | + | + | + | + | + | + | + | - | + | - | + |
| 4 | 52 | M | + | + | - | - | - | - | + | - | - | + | + | + | + | + | + | + | - | - | + | + |
| 5 | 58 | F | + | - | - | - | - | - | + | - | - | + | + | + | + | + | + | + | - | + | + | + |
| 6 | 36 | F | + | - | - | + | - | + | - | - | - | + | + | + | + | + | + | + | - | + | - | + |
| 7 | 60 | F | + | - | - | + | + | - | - | - | + | + | + | + | + | + | + | + | - | + | + | + |
| 8 | 65 | F | + | - | + | - | - | - | - | - | - | + | + | + | - | + | + | + | + | - | - | + |
| 9 | 59 | M | + | + | + | - | - | + | + | + | + | + | + | + | + | + | + | + | + | - | - | + |
| 10 | 56 | M | + | + | + | + | + | - | - | - | + | + | + | - | + | - | + | + | - | + | + | - |
| 11 | 70 | M | + | + | - | + | + | - | - | - | - | + | + | + | + | + | + | + | - | + | + | + |
| 12 | 24 | F | + | + | - | + | - | - | + | - | - | + | + | + | + | + | + | + | - | + | - | + |
| 13 | 64 | M | + | + | + | + | + | - | - | - | + | + | + | - | + | - | + | + | - | + | + | - |
| 14 | 74 | F | + | + | + | + | + | - | - | - | - | + | + | - | + | - | + | + | - | + | + | - |
| 15 | 59 | M | + | + | + | + | + | - | - | - | - | + | + | - | + | - | + | + | + | + | + | - |
| 16 | 70 | F | + | - | - | - | + | - | + | - | - | + | + | + | + | + | + | + | - | - | + | + |

**Supplementary Table 1. Clinical manifestations, Japanese Ministry of Health, Labor and Welfare criteria items and** **classification criteria of the American College of Rheumatology criteria at diagnosis**

Japanese Ministry of Health, Labor and Welfare criteria：① (A)+(B)+(C)+(D), ②(A)+(B)+(C)+(E) ① or ② definite, American College of Rheumatology criteria：4/6 items definite

ENT; Ear, Nose, Throat
