# Supplementary material for: Effectiveness and safety of mepolizumab in combination with corticosteroids in patients with eosinophilic granulomatosis with polyangiitis
Source: Arthritis Res Ther. 2021 Mar 16;23:86. doi: 10.1186/s13075-021-02462-6 (PMC7962235; doi:10.1186/s13075-021-02462-6)
Supplement: Supplementary file 2 — Additional file 2: Supplementary Table 2. Baseline characteristic of 16 Patients with Eosinophilic Granulomatosis with Polyangiitis. [file 13075_2021_2462_MOESM2_ESM.docx]

| Case  No. | age | disease  duration  (month) | treatment history | Relapsing /Refractory | CS dose PSL  (mg/day) | Steroid treatment period | Immuno-suppressants | BVAS | BAVS items(score) | VDI | VDI items | ANCA status | Absolute  eosinophil count(/μL) | Serum IL-5 concentration  (pg/mL) | CRP (mg/dL) |
| --- | --- | --- | --- | --- | --- | --- | --- | --- | --- | --- | --- | --- | --- | --- | --- |
| 1 | 63 | 144 | high-dose CS, AZ, MTX, IVCY, IVIG | Relapsing  (CS dose increasing) | 15 | 144 | none | 0 |  | 7 | Chronic bronchial asthma, chronic respiratory failure, peripheral neuropathy, abnormal respiratory function tests, myocardial infarction, infiltration shadow, pleural effusion | - | 0 | 0.27 | 0.89 |
| 2 | 57 | 176 | high-dose CS, AZ, MTX | Relapsing  (BVS score worsening) | 5 | 176 | AZ, MTX | 3 | persistent sinusitis(1), asthma(2) | 5 | Chronic bronchial asthma, abnormal respiratory function tests, chronic sinusitis, dyslipidemia, Chronic bronchitis | - | 117 | 0.12 | 0.03 |
| 3 | 36 | 22 | high-dose CS, AZ, IVCY | Relapsing  (BVS score worsening) | 2.5 | 22 | AZ | 2 | asthma worsening(2) | 3 | Chronic bronchial asthma, abnormal respiratory function tests, chronic sinusitis | - | 1950 | 4.86 | 0.09 |
| 4 | 52 | 96 | high-dose CS, AZ | Relapsing  (BVS score worsening) | 12 | 96 | none | 3 | erythema worsening(2),  arthralgia worsening(1) | 3 | Chronic bronchial asthma, Persistent arthritis, chronic erythema | - | 6639 | 36.33 | 0.13 |
| 5 | 69 | 144 | high-dose CS, AZ, MTX,  IVCY, IVIG | Refractory  (CS dose >4mg/day) | 7 | 144 | AZ, MTX | 0 |  | 4 | Chronic bronchial asthma, chronic sinusitis, abnormal respiratory function tests, Nasal congestion | - | 60 | 0.31 | 0.05 |
| 6 | 50 | 168 | high-dose CS, AZ, MTX, IVCY | Relapsing  (BVS score worsening) | 0 | 168 | MTX | 2 | asthma worsening(2) | 4 | Chronic bronchial asthma, abnormal respiratory function tests, Cardiomyopathy, deafness | - | 4872 | 9.02 | 0.27 |
| 7 | 61 | 13 | low-dose CS | Relapsing  (BVS score worsening) | 0 | 13 | none | 5 | persistent asthma(1),  infiltrate worsening(4) | 3 | Chronic bronchial asthma, chronic sinusitis, Diabetes mellitus | + | 1496 | 2.36 | 0.07 |
| 8 | 67 | 25 | high-dose CS, AZ, MTX,  IVIG | Refractory  (CS dose >4mg/day) | 6 | 25 | MTX | 0 |  | 3 | Chronic bronchial asthma, peripheral neuropathy, dyslipidemia | - | 210 | 1.73 | 0.01 |
| 9 | 62 | 40 | high-dose CS, AZ, IVCY | Relapsing  (BVS score worsening) | 3 | 40 | AZ | 6 | persistent worsening(2),  infiltrate worsening(4) | 6 | Chronic bronchial asthma, abnormal respiratory function tests, Cardiomyopathy, peripheral neuropathy, Diabetes mellitus, Osteoporosis | - | 18318 | 14.90 | 1.92 |
| 10 | 57 | 15 | CS pulse, high-dose CS,  IVCY | Refractory  (CS dose >4mg/day) | 20 | 15 | none | 0 |  | 4 | Chronic bronchial asthma, peripheral neuropathy, Osteoporosis, Low vision | - | 0 | 14.67 | 0.06 |
| 11 | 75 | 60 | high-dose CS, AZ, TAC,  MTX, IVCY | Relapsing  (BVS score worsening) | 7 | 60 | TAC | 6 | persistent worsening(2),  infiltrate worsening(4) | 7 | Chronic bronchial asthma, chronic sinusitis, abnormal respiratory function tests, peripheral neuropathy, Cardiomyopathy, Osteoporosis, Diabetes mellitus | - | 108 | 0.62 | 0.08 |
| 12 | 30 | 72 | high-dose CS, AZ, IVCY,  RTX | Refractory  (CS dose >4mg/day) | 10 | 72 | none | 0 |  | 3 | chronic sinusitis, chronic bronchitis, deafness | - | 156 | 2.02 | 0.15 |
| 13 | 78 | 168 | high-dose CS, AZ, IVCY,  IVIG | Relapsing  (BVS score worsening) | 10 | 168 | AZ | 0 |  | 6 | chronic sinusitis, chronic bronchitis, peripheral neuropathy, deafness,  Osteoporosis, Diabetes mellitus | - | 0 | 0.36 | 0.03 |
| 14 | 78 | 48 | CS pulse, high-dose CS,  AZ, IVIG | Refractory  (CS dose >4mg/day) | 5 | 48 | none | 0 |  | 4 | Chronic bronchial asthma, abnormal respiratory function tests, peripheral neuropathy, Osteoporosis | - | 376 | 0.04 | 0.13 |
| 15 | 61 | 22 | high-dose CS, IVIG | Refractory  (CS dose >4mg/day) | 7 | 22 | AZ | 0 |  | 3 | chronic bronchitis, peripheral neuropathy, hpertension | - | 60 | 0.16 | 1.67 |
| 16 | 71 | 16 | low-dose CS | Relapsing  (BVS score worsening) | 0 | 16 | none | 4 | sinusitis worsening(2),  purpura worsening(2) | 0 |  | - | 2655 | 8.74 | 0.05 |

**Supplementary Table 2. Baseline characteristic of 16 Patients with Eosinophilic Granulomatosis with Polyangiitis**

CS: corticosteroid(prednisolone or equivalent), IVCY: cyclophosphamide pulse therapy i.v., RTX: rituximab, MTX: methotrexate, AZ: azathioprine, TAC: tacrolimus, BVAS: Birmingham Vasculitis Activity Score. VDI: Vasculiis damage index
