# Supplementary figures and images for: Effectiveness and safety of mepolizumab in combination with corticosteroids in patients with eosinophilic granulomatosis with polyangiitis
Source: Arthritis Res Ther. 2021 Mar 16;23:86. doi: 10.1186/s13075-021-02462-6 (PMC7962235; doi:10.1186/s13075-021-02462-6)

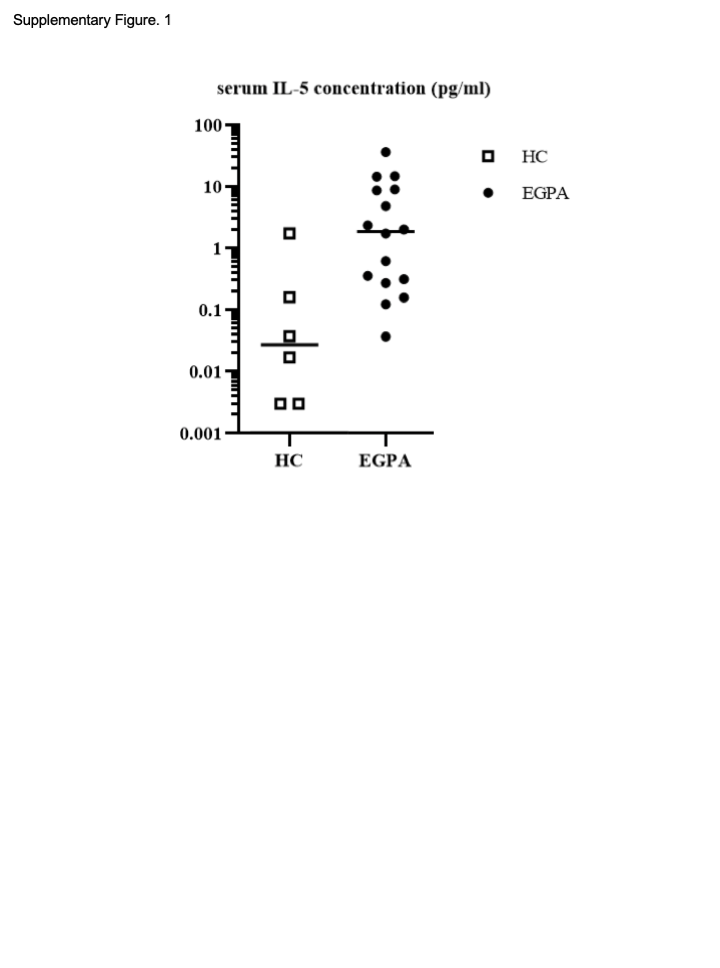

Supplement: Supplementary file 3 — Additional file 3: Supplementary Fig. 1. Serum IL-5 concentration of 16 Patients with EGPA before initiating MPZ and health controls (HC group). P values were determined by Mann-Whitney’s U test. p* < 0.01: EGPA group (n = 16) vs. HC (n = 6). [file 13075_2021_2462_MOESM3_ESM.tiff]
